# Semantic composition of robotic solver algorithms on graph structures

**DOI:** 10.3389/frobt.2024.1363150

**Published:** 2025-01-29

**Authors:** Sven Schneider, Nico Hochgeschwender, Herman Bruyninckx

**Affiliations:** ^1^ Department of Computer Science, Institute for AI and Autonomous Systems, Hochschule Bonn-Rhein-Sieg, Sankt Augustin, Germany; ^2^ Department of Mechanical Engineering, KU Leuven, Leuven, Belgium; ^3^ Department of Mathematics and Computer Science, University of Bremen, Bremen, Germany; ^4^ Department of Mechanical Engineering, TU/e Eindhoven, Eindhoven, Netherlands; ^5^ Flanders Make, Lommel, Belgium

**Keywords:** solvers based on graph traversal, model-based engineering, algorithm synthesis, code generation, composability and compositionality, kinematics and dynamics

## Abstract

This article introduces a model-based design, implementation, deployment, and execution methodology, with tools supporting the systematic composition of algorithms from generic and domain-specific computational building blocks that prevent code duplication and enable robots to adapt their software themselves. The envisaged algorithms are numerical solvers based on graph structures. In this article, we focus on kinematics and dynamics algorithms, but examples such as message passing on probabilistic networks and factor graphs or cascade control diagrams fall under the same pattern. The tools rely on mature standards from the Semantic Web. They first synthesize algorithms symbolically, from which they then generate efficient code. The use case is an overactuated mobile robot with two redundant arms.

## 1 Introduction

**FIGURE 1:**
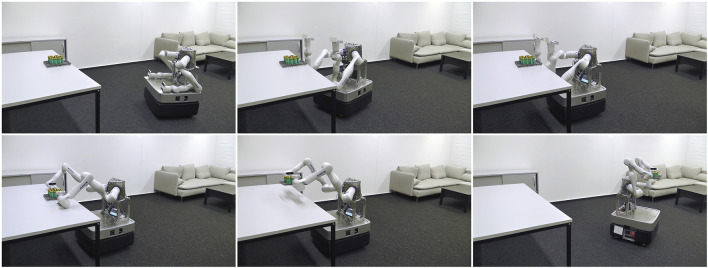
Complicated robotic system solving a dual-arm manipulation task. In the first row, the robot approaches the table with its mobile base to then perform a touch-based alignment. In the second row, the two manipulators grasp and lift the object. Finally, the robot leaves the table while carrying the object.

Figure 1 shows a complicated, overactuated mobile robot with two redundant, torque-controlled arms performing a dual-arm manipulation task. A typical implementation of such an application relies on a wide range of algorithms, including (i) kinematics and dynamics solvers for forward kinematics or inverse dynamics problems as available in libraries like Pinocchio (Carpentier et al., 2019), the Rigid Body Dynamics Library (RBDL) (Felis, 2016), or the Kinematics and Dynamics Library (KDL)^1^; (ii) probabilistic filters and estimators, implemented by libraries such as the Georgia Tech Smoothing and Mapping library (GTSAM) (Dellaert, 2012), or the Bayesian Filtering Library (BFL)^2^, to determine the state of the robot and its environment, for example, by simultaneous localization and mapping (SLAM); (iii) data-flow computations in cascade control diagrams such as the MATLAB Control System Toolbox^3^, in the Stack-of-Task’s (Mansard et al., 2009) dynamic-graph^4^, or in video-processing pipelines like GStreamer^5^; and (iv) task specifications, expressing the *desired* behavior of the robot’s dynamics and its controllers as well as the *desired* sensor processing outputs, realized via expression graphs (Aertbeliën and De Schutter, 2014). The overall integration of such functionalities and their realization that the robot requires to solve its tasks is also known as a robot control architecture.

Even if these algorithms and libraries originate from different yet highly relevant robotics domains, they share two important commonalities. First, they rely on an underlying structural model of a *graph* that represents a kinematic chain, a probabilistic network or factor graph, a data-flow network between operators, and an expression graph, respectively. Second, they answer *queries* on these graphs by (i) *propagating* data between the graph’s nodes and (ii) *dispatching* ^6^ computations on that data for each visited node or edge while (iii) performing one or more graph *traversals*. Here, a traversal represents a particular choice of *serializing* or *scheduling* the computations to establish a computational control flow. The good news is that for many of these queries, the knowledge already exists about how to create efficient execution schedules. This includes kinematics and dynamics problems (Popov et al., 1978), inference in Bayesian networks (Pearl, 1982), or cutting cascade control loops into a series of computations for each time scale. The main differences between these solvers comprise the data encoded in the graph and the specific policies or choices imposed on the algorithms, that is, which data to propagate, which computations to perform, and how to traverse the graph. The solution to such a recurring problem in architectures is known as a *design pattern* and has been popularized in software engineering by the “Gang of Four” (Gamma et al., 1994) in object-oriented software development. Hence, given these commonalities and differences, we classify such *graph-based solvers* as a fundamental pattern that has not yet been described in the existing literature.

On the one hand, software libraries that implement graph-based solvers allow their users to customize the structural graphs at compile time. On the other hand, they keep the solver algorithms that act on these graphs inaccessible, which leads to the following three problems. First, such designs prevent many customizations and optimizations of the computational control flow as well as the introspection and instrumentation of the executing algorithms. The easy way to introduce a new algorithm or adapt an existing one is for developers to implement it completely from scratch or by copying and refactoring a previous implementation. For instance, in KDL, we have counted twelve realizations of the computations for the forward position kinematics (FPK) and seven realizations for the forward velocity kinematics (FVK) across 22 solvers in total. This is a clear violation of the “Don’t Repeat Yourself” (DRY) (Hunt and Thomas, 2019) principle for good software engineering and leads to technical debt. A second problem comes from how the libraries support *configuration*. One approach is to create an application programming interface (API) where the configuration options are part of the input parameters. This leads to very long function signatures, so the pragmatic choice is to limit the configuration capabilities of the library. Another (better) approach to configuration is to provide a *setters and getters* API via which any desired set of parameters can be given new values at runtime. However, this introduces the risk of data inconsistencies because, in most cases, several parameters should be updated *together* in an *atomic* way. A third problem is that, at runtime, applications may require multiple solvers with partially overlapping “computational states,” such as the position and motion of sensors or tools on the robot’s kinematic chain. This leads to redundant computations and challenges in keeping a consistent state between these multiple solvers.

To address these problems, this article introduces the *software engineering* aspects of a model-based design, implementation, deployment, and execution methodology, with tools supporting the systematic composition of algorithms from generic and domain-specific computational building blocks. A first contribution is that the granularity of these building blocks is designed for **composability**: on the one hand, they are so small that each of them is a pure function and, on the other hand, they need not be smaller than what is contained in one node of the graph that represents the computational control flow. That is, where the if-then-elses and the for or while loops are introduced to differentiate between different behaviors of the executed algorithms. The algorithm’s building blocks are models of the *data structures*, the pure *functions* that act on this data, and the order in which these functions are called, that is, the *schedule* or *control flow*. We present the *mechanism* to model, compose, and execute complicated algorithms. Simultaneously, we ensure that each mechanism is *configurable* so that a large variety of data-flow *policies* can be composed on top. Examples include, among others, incremental computations by processing sub-graphs on demand, employing optimized computations for sub-graphs, or injecting instrumentation and logging into the algorithm. In summary, our main contributions are:

• We analyze kinematics and dynamics solvers as the main representatives of algorithms that perform computations on graph structures. Their commonalities and differences allow us to identify and describe the graph-based solver pattern.
• We derive free and open-source licensed, vendor-neutral models and metamodels^7^ to represent and compose graph-based solvers for kinematics and dynamics solvers. The models include the data structures, the operators or functions that manipulate these data structures, and the ordering constraint on the functions. We reify each of these elements so that they can be symbolically referenced.
• We develop a toolchain that processes the above graph-structured models using symbolic queries to synthesize kinematics and dynamics solvers and generate code from the resulting models. We complement the toolchain by an implementation of a software library that implements the pure functions to implement the solvers. Both are released under a free and open-source license.
• We showcase the models, toolchain, and backend software library in a case study for kinematics and dynamics solvers.

The remainder of this article is structured as follows. In Section 2, we revisit the application to provide a detailed review of kinematics and dynamics solvers to then derive requirements for graph-based solvers in Section 3. Afterward, Section 4 provides the background on composable models. We present the tooling for solver synthesis and code generation in Section 5, followed by a case study in Section 6. Section 7 discusses our approach and tools, while Section 8 concludes the article.

## 2 Kinematics and dynamics solvers

This section describes the structural and computational policies used in numerical solvers for kinematic chains via (i) the topology of the underlying graphs; (ii) the types of traversals (that is, the serialization of the computations) over these graphs; (iii) the representation of data structures; (iv) the types of computations on these data structures; (v) the handling of cycles in the graphs; (vi) the handling of domain-specific, composite and hierarchical nodes; and (vii) the support of incremental computations to only evaluate output that depends on changed input and caching of intermediate results. In the supplementary material, we provide an additional analysis of graph-based solvers for probabilistic networks, data-flow programming, and expression graphs.

**FIGURE 2:**
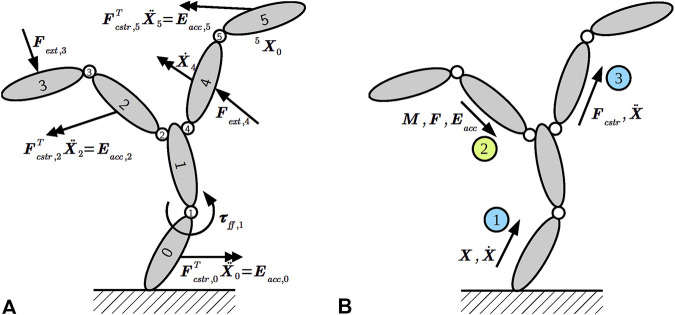
**(A)** Kinematic chain with three types of motion drivers attached to links and joints as solver input: desired Cartesian accelerations via constraint forces $F_{\mathrm{cstr}}$ and acceleration energy $E_{\mathrm{acc}}$ ; Cartesian external forces $F_{\mathrm{ext}}$ ; and joint torques $\tau_{ff}$ . The solver’s output is the answer to the query that asks for the fifth link’s pose ^5^ $X_{0}$ and the fourth link’s velocity ${\dot{X}}_{4}$ . **(B)** Kinematic chain consisting of links and joint with positions X and velocities $\dot{X}$ propagated outward (blue circle) during the first sweep; inertia M , force F , and acceleration energy $E_{\mathrm{acc}}$ propagated inward (green circle) during the second sweep; as well as constraint forces $F_{\mathrm{cstr}}$ and acceleration $\ddot{X}$ propagated outward during the third sweep.

The robot in Figure 1 exemplifies the most relevant types of kinematic chains: each of the two manipulators by itself is a *serial chain*, but when connecting both arms to the robot, a *tree-structured chain* with the torso as its root emerges. Finally, the mobile base is an example of a *parallel chain* where the ground couples (or “constrains”) the motion of all wheels. The objective of solvers that act on such kinematic chains is to answer queries that compute the instantaneous forces and motions of all links when a particular subset of them is given as inputs (or “drivers”) for the motion. Figure 2A depicts an example where multiple motion drivers are attached to a kinematic chain to move or accelerate a body in a certain direction while resisting external forces. Additionally, the application specifies the expected solver outputs, such as the pose (position and orientation) and velocity of an end-effector, or the joint-level control torques to achieve the desired motions.

The following paragraphs provide some concrete examples of queries and their solvers. Algorithm 1 shows an FPK solver that, given a model of a kinematic chain with N bodies and the joint positions q as inputs, computes the pose ^*i*^ $X_{0}$ of each body i with respect to the root body 0. To this end, in Line 2, it composes the static pose over the body (or “link”) $X_{L,i}$ with the pose over the joint $X_{J,i}\left(q_{i}\right)$ that depends on the current joint position. The result is the relative pose of the current body i with respect to its parent p(i) . Then, in Line 4, the solver accumulates the parent’s pose with that relative pose. Here, a single outward traversal (Line 1) of the kinematic chain from a selected root to the leaves suffices to compute the answer. In the context of kinematic chains, such a graph traversal that serializes kinematic or dynamic computations is also called a *sweep*.

Algorithm 1 Forward position kinematics.

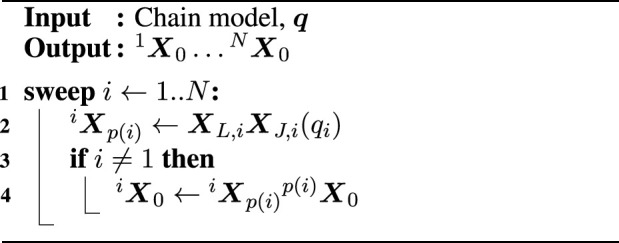

The FVK solver in Algorithm 2 computes the Cartesian velocity ${\dot{X}}_{i}$ for each body in the kinematic chain given the joint velocities $\dot{q}$ . A comparison of the FVK solver with the FPK solver reveals that the former is an extension of the latter: only two lines have been added, while the others remain the same. These two lines are the initialization of the root body’s velocity (Line 1) and the accumulation of velocities along the kinematic chain (Line 6). The accumulation step consists of, from right to left, (i) mapping the joint velocity ${\dot{q}}_{i}$ to Cartesian space with the joint Jacobian $S_{i}$ ; (ii) transforming that Cartesian velocity to the root coordinate frame using the inverse transformation matrix ^*i*^ $X_{0}$ ; and (iii) adding the Cartesian velocity ${\dot{X}}_{p\left(i\right)}$ that has already been accumulated in the previous step, up to and including the parent body.

Algorithm 2 Forward velocity kinematics.

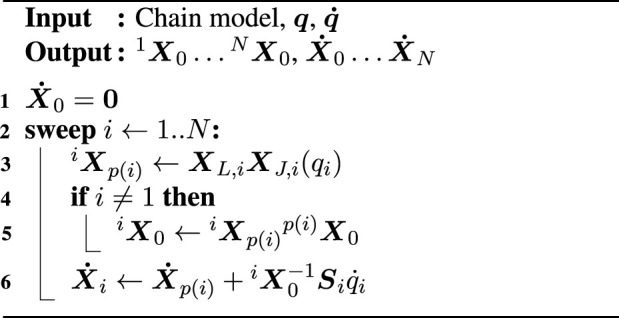

Another policy for the velocity accumulation step is to express the velocities in the moving coordinate frame instead of the stationary root frame: X˙i←Xp(i)iX˙p(i)+Siq˙i . *Many* more such policies exist, especially when considering complicated algorithms, including forward and inverse dynamics solvers (cf. Featherstone, 2008; Vereshchagin, 1989) that map forces to accelerations and *vice versa*, respectively. The variety in solver policies is due to the large set of choices that are possible, for example, (i) the choice of physical units that must be kept consistent across all operations; (ii) the propagation of the motion drivers that could either be accumulated as soon as possible (for the most efficient computations) or only during the third, solver sweep (for most flexibility); or (iii) the choice of matrix inversion and the handling of singularities during such an inversion. The two solver examples already demonstrate how a naïve implementation of such algorithms leads to code duplication when each algorithm resides in its own function or class, as is commonly the case in software libraries. For example, a hypothetical solver library may provide the functions fpk(chain, q) for Algorithm 1, fvk_stationary(chain, q, qd) for Algorithm 2, and fvk_moving(chain, q, qd) for the choice of the moving coordinate frame, each containing computations of the FPK solver. The number of policies increases even further when the chain’s dynamics also enter into the solver. 

The two algorithms above demonstrate a computation *on* a graph. The graph represents the kinematic and dynamic properties of the kinematic chain (including the *topology* of the connections between links) but does not contain all data structures found in the algorithms. Instead, all variables apart from the already specified pose over the link, $X_{L,i}$ , must be added to the graph. The computations are the various types of operators with *physical* meaning that are represented *mathematically* by either matrix multiplication or vector addition (composition of poses, maps from joint space to Cartesian space, transformation of a velocity, or addition of two velocities). The top-to-bottom order of the lines is a physically imposed ordering constraint: here, the transformation of velocities depends on (the presence of) poses. Finally, more complicated solvers rely on up to three sweeps, as depicted in Figure 2B: positions and velocities travel outward from the root to the leaves in the first sweep; inertia, force, and acceleration energy travel inward in the opposite direction during the second sweep; the third sweep is outward again, accumulating those computational results that are needed for the actual query.

Some kinematic chains have *cycles*. Solvers deal with such cycles in two complementary ways. The first option is to cut an edge in each cycle, which results in a spanning tree of the kinematic chain. For each cut, the solver adds Cartesian acceleration constraints on either side of the cut, representing the physical reality that both sides of the cut must move with the same acceleration. That reality is a *constraint* that the solver algorithm must take into account. The solver deals with the loop constraint by computing the *constraint forces* that would make both sides of the cut accelerate in exactly the same way. The second option is to cluster the cycles into composite nodes that must then be solved for numerically using an explicit matrix inversion (Jain, 2012; Chignoli et al., 2023).

We have already introduced a hierarchy in the description of the types of kinematic chains above. Here, the apex of that hierarchy could be the whole robot, the base with two arms, to be treated as one kinematic chain on which a single solver operates. However, more commonly, roboticists decouple the arms from the base and associate dedicated solvers with each; often, kinematics solvers that run at lower control frequencies suffice for the base, whereas for the arms dynamics solvers at higher control frequencies are required to handle contact situations. Finally, the individual joints of the arms are the “smallest” kinematic chains. Yet, even at this level, joints could actually be composites. As an example, one model of a spherical joint is a sequence of three revolute joints. Then, solvers dispatch specialized computations depending on the joint type. For example, one-dimensional joints such as revolute and prismatic joints allow for computationally efficient solutions that rely on scalars instead of full matrices.

We have presented an example of incremental computations in dynamics solvers in Schneider and Bruyninckx (2019): the propagation of the so-called articulated-body inertia matrix (see Featherstone, 2008) is a computationally expensive operation. Additionally, the inertia matrix does not change significantly in neighboring configurations, while its parameterization is prone to measurement noise. Consequently, it is a good candidate for a computation that is performed at a reduced frequency in comparison to the propagation of the other quantities. The articulated-body inertia matrix is then cached and reused across multiple solver invocations.

## 3 Requirements for graph-based solvers

Physical and scientific constraints exist that lead to efficient solvers for kinematic chains and graphical models. It is the top-level tree structure of the underlying graphs that enables the application of dynamic programming. For graphs with cycles, the graph must be pre-processed to establish a tree-structured *view* on the graph, either as a spanning tree or a hierarchical decomposition as in the junction tree algorithm. On the one hand, dynamic programming dictates which data structures should be cached at each node and which operations should be performed on that data. On the other hand, it coordinates or schedules the computations along the graph traversal. Two sweeps, one inward and one outward, decompose the graph’s state that can then be flexibly and efficiently recomposed in a final solver sweep to answer queries. The scheduling can depend on various types of state or runtime conditions, such as the availability of data or conflicts in the motion specification. Hence, we can encode such a solver algorithm as a computational graph on top of the underlying structural graph. The latter is the basis of the former’s *bookkeeping* (which data structures to use in which operations), *configuration* (which values to fill into the data structures), and *coordination* (in which order to execute the operations). For data-flow networks and expression graphs, these three points are completely at the developers’ disposal, who must rely on their insights into the domain to design the algorithms. Nevertheless, the same algorithmic building blocks exist in these approaches.

We can derive various requirements for our approach from the analysis. First, we need explicit models of the graphs’ structure and of how behavior is attached to that structure. Here, behavior refers to explicit models of algorithms that consist of data structures, functions, and schedules. The various computational policies, such as caching of intermediate results or varying execution frequencies are then a higher-order composition to the algorithms. A second necessity is flexible tooling that efficiently synthesizes the domain-specific algorithms and attaches them to the underlying graph models. Because the algorithms are merely models, additional tools are required that can execute these models by interpretation or compilation. Finally, all of the above models should be unambiguously understandable by a robot so that it can automatically adapt its software, also at runtime.

## 4 Composable and compositional models for kinematic chains

In this section, we summarize the main results of our prior work from Schneider et al. (2023) to represent the above-mentioned graphs (their structure and their “behavior”) as they are a prerequisite for the remainder of this article. In that publication, we have presented an in-depth analysis of existing modeling formats, including the Unified Robot Description Format (URDF)^8^ and the Semantic Robot Description Format (SRDF)^9^ that originate from the Robot Operating System (ROS) ecosystem (Quigley et al., 2009). Given the lessons learned, we have designed and realized composable and compositional models in JSON-LD (Sporny et al., 2020). **Composability** pertains to *structure* and is an application of two major software design principles to models. The first is the *open-closed principle* (Meyer, 1997), which implies that it should always be possible to extend existing models without a need for modification. The second is the *single-responsibility principle* (Martin, 2003), which implies that each model should represent exactly one concern. In relational databases, the latter principle is known as the third normal form (Codd, 1971; Kent, 1983): each table has a single “topic” and only contains direct dependencies on the table’s key; that is, it only represents intrinsic *properties* instead of extrinsic *attributes* (Bruyninckx, 2023, Section 1.5.3). **Compositionality** is concerned with model *semantics*. It implies that each model must have an unambiguous meaning^10^. For composite models, that meaning must follow from the meaning of its constituents and the composition rules (which are *higher-order models*, that is, a set of relations with other models as arguments) so as to avoid unpredictable “emergent” behavior of the composed system. Both these design goals, composability and compositionality, are highly relevant in complex modern robots that act in open environments and open-ended missions where (i) designers usually cannot foresee all possible applications of their models and, hence, should avoid introducing artificial limitations; and (ii) robots must be able to interpret and reason about the models by themselves without having to rely on human developers to transform the models to code.

JSON-LD models are both JavaScript Object Notation (JSON) (Bray, 2017) documents and Resource Description Format (RDF) (Cyganiak et al., 2014) documents^11^. They support composability and compositionality because all model elements (i) have unique identifiers so that they can be referenced from “external” sources such as files on servers or even executing software binaries; (ii) can refer to complete metamodels that unambiguously define the models’ semantics, so that they are free from implicit assumptions; and (iii) are loosely coupled due to the underlying, generic graph structure as well as the support for “symbolic pointers” that are represented by Internationalized Resource Identifiers (IRI) as defined by Duerst and Suignard (2005). In the following subsections, we introduce concrete JSON-LD models of kinematic chains and their behavior as a running example. The proper design of the underlying metamodels can only originate from a detailed and exhaustive domain analysis as we have performed for kinematic chains here and for the additional domains in the supplementary material. As a typographic convention, we indicate model elements in a monospaced font. In addition, we designate models by concise and human-understandable identifiers, yet their real meaning must only come from their properties and metamodels.

**FIGURE 3:**
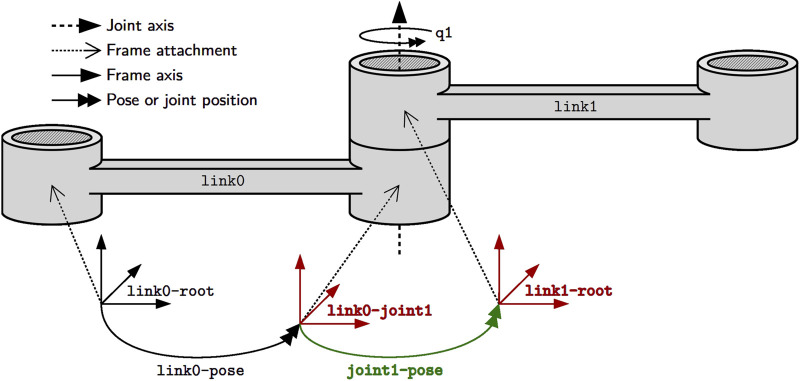
The joint joint1 constrains the relative motion of the two frames link0-joint1 and link1-root that are associated with the links link0 and link1, respectively. For example, the pose joint1-pose of the latter frame with respect to the former frame is associated with the joint position q1 and can change over time. Finally, pose link0-pose locates the joint frame on link link0 with respect to the root frame link0-root. For rigid bodies, this pose remains static.

Figure 3 depicts two links that are constrained in their relative motion by a joint. We consider the most abstract representation of a link or body, its “skeleton,” as simply a collection of simplices, that is, geometric entities such as points, lines, or frames. These simplices are attachment points for, among others, shape geometry, inertia, motion specifications and also joints as textually represented using JSON-LD in Listing 1. Syntactically, JSON-LD can add one identifier (@id keyword), one or more types (@type keyword), and one context (@context keyword) to any JSON object. The *referenced context*, as a list of IRIs, symbolically points to all *metamodels* that define the meaning of this model. Part of that metamodel is the structural constraint that the Joint type demands the between-attachments property, as indicated by the matching colors. One way to formally represent such a constraint is via the Shape Constraint Language (SHACL) defined in Knublauch and Kontokostas (2017). Another part of the metamodel defines that the between-attachments property symbolically refers to a list of all simplices that are, on the one hand, attached to bodies and, on the other hand, are involved in the joint-constraint relation. Similar to the body, this is the most abstract representation of a joint that captures nothing more than the joint’s constituents. The type of joint (e.g., revolute or prismatic), its geometric constraints (e.g., a revolute joint keeps two lines attached to both bodies coincident), or its direction of motion must be composed on top of this model as indicated by the ellipsis. More complicated kinematic chains are represented by ordered collections of joints. Thus, our models can represent kinematic chains of arbitrary topology: serial, tree-structured, and parallel (that is, with one or more cycles).

Listing 1 Textual model of joint rob:joint1. Colors that match with Figure 3 indicate identical entities.

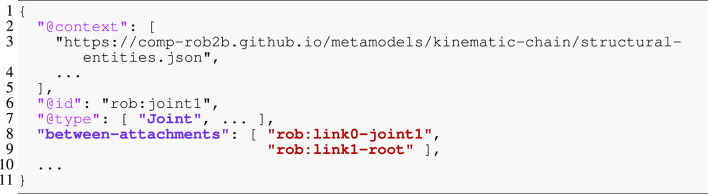

The pose in Listing 2 is a relation over the *same* frames as those present in the joint relation to represent the coordinate-free position and orientation of the joint’s constrained motion. In the context of a Pose, these two frames play the role of an of frame and a with-respect-to (or wrt) frame, respectively. Listing 3 then introduces concrete coordinates in 3D-Euclidean space (as a unitless direction-cosine matrix and a position vector measured in meters). This model shows an example of *multi-conformance*, meaning that an entity can have more than one type to define its semantics, a feature that is rarely encountered in modeling approaches or general-purpose programming languages. Moreover, JSON-LD helps in distinguishing properties by mapping them to IRIs: the of property in Listing 2 Line 5 has a different meaning from the one in Listing 3 Line 11. To this end, the *embedded context* maps the latter to the IRI coord:of-pose (Line 5), where coord is the prefix (or “namespace”) defined in Line 3. Additionally, this context defines the of property as a symbolic pointer (Line 6). We again notice the recurrence of the same rob:link0-joint1 frame in Line 12 that we have already encountered above.

Listing 2 Pose relation in JSON-LD.

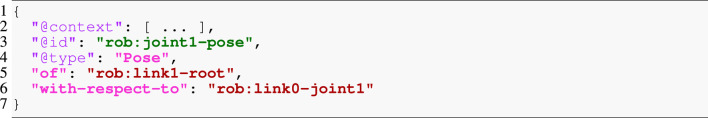

The model of a function (or operator) follows the same pattern: it features an identifier, a type, and its properties, as exemplified in Listing 4. The semantics are defined in the metamodel that is referenced by the type. In this example, the operator represents a map from a joint-space position rob:q1 to a pose rob:joint1-pose in Cartesian space. The metamodel also imposes structural constraints, for example, that the joint position and the pose are associated with the same joint. There are two noteworthy remarks. First, the model *represents* an operator but does not “execute” it; instead, that evaluation is the result of a model transformation via some interpretation or compilation. Second, multiple instances of the same operator, that is, operators with the same type, can exist. In that sense, when compared with general-purpose programming languages, the type defined in the metamodel resembles a function declaration, whereas an instance establishes the connection or binding of data structures, similar to a *closure* in functional programming languages. The execution or invocation of such a function is represented by an entity of type Schedule with a single property trigger-chain ^12^, an ordered list of symbolic pointers to operators.

Our approach generalizes the geometric relations semantics (GRS) (De Laet et al., 2012) in two ways. Although the GRS do separate the coordinates from their coordinate-free relation, they do not reify the latter. Here, instead, we assign unique identifiers to both representations, which enables us to properly express the one-to-many relations from the former to the latter. Furthermore, we extend the GRS to the models of kinematic chains and to the dynamics solvers on top. This includes physical quantities such as acceleration, force, and inertia together with their operators. Having symbolic models of kinematic chains allows pre-processing or “normalization.” This includes, for example, the extraction of a spanning tree, the conversion of all quantities to matching physical units, the composition of static chains of pose relations, or the transformation of inertia to frames that are most suitable for the solvers.

Listing 3 Pose coordinate representation.

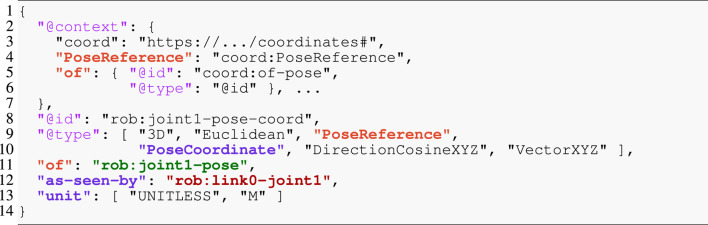

Listing 4 Forward position kinematics operator of a joint.

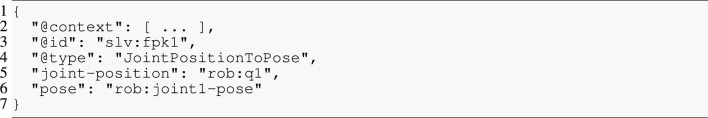

## 5 Tooling implementation: synthesizer and code generator

**FIGURE 4:**
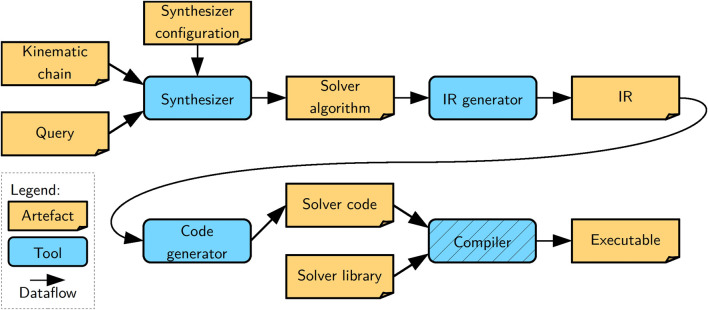
High-level architecture of our toolchain showing the three developed tools (blue boxes without hatching), a general-purpose compiler (blue box with hatching), and the artifacts (orange boxes) they consume or produce (arrows).

In this section, we describe our tooling **to synthesize** concrete algorithm models for kinematics and dynamics solvers and **to generate** correct-by-construction code from such models. Synthesis entails deriving data structures, function instances, and a schedule. The generated code can then be seen as a dispatcher of that schedule. Figure 4 depicts the architecture of our toolchain, which consists of three main tools. The *synthesizer* that consumes a model of a kinematic chain, a query model composed on top of that kinematic chain, and a dedicated solver configuration or a “template” of the solver. It produces as output an algorithm model that can be seen as an instantiation of the template along the kinematic chain. This algorithm is fully linked to the kinematic chain model, meaning it is a graph that symbolically points to elements of the kinematic chain. The *IR generator* lowers the algorithm model to an intermediate representation (IR) that the template-based *code generator* then transforms to code in a general-purpose programming language. It is a best practice to keep any logic out of the code generator. Hence, the IR generator performs any pre-processing required for the code generator. Thus, lowering entails the preparation of the algorithm for the code generator by serializing the graph to a tree and introducing any necessary transformations. Finally, given software libraries that provide the pure solver functions, this code is compiled into an executable with a general-purpose compiler. We provide more details on the implementation of all tools and models in the following discussion.

### 5.1 Synthesizer

The overall process of synthesis is a form of **graph rewriting**, that is, matching patterns in the graph and replacing them with new patterns. In general, due to the subgraph isomorphism problem, this is an NP-complete problem (Cook, 1971). However, we can exploit domain-specific knowledge that enables us to (i) guide the traversal over the graph structure; and hence (ii) reduce graph matching on the overall graph to a local neighborhood or even simply localized graph traversals.

We have implemented the synthesizer using the established RDFLib^13^ Python library, which also supports the standardized, powerful, and mature graph query language SPARQL (Harris and Seaborne, 2013). The step change in employing this setup is that (i) SPARQL enables the declarative formulation of complicated graph matching and even graph rewriting queries; (ii) in SPARQL the directionality of edges does not constrain traversability so that a query can follow edges in the “opposite” direction, (iii) RDFLib allows “anchoring” these queries in the underlying graph to drastically improve performance by restricting the graph matching to the above-mentioned neighborhood of these “anchor points,” and (iv) RDFLib provides a tight interface of custom code with the queries.

The synthesizer features a modular architecture with a small framework core that is complemented by modules to realize the interaction with the graph, for example, by emitting the required data structures and operators for performing the FPK computations. Inspired by the terminology of Gremlin (Rodriguez, 2015), we call each module a *step*. A step *declares* to the framework (i) an expansion query that, on the one hand, determines where the traversal through the graph should continue and, on the other hand, is a first filter criterion to determine when the step applies; and (ii) the functions that implement the graph manipulations at the nodes (either the parent and child or only the child) reached by the expansion. The pure declaration has the benefit that the framework can pre-process and optimize the query execution. Specifically, we have noticed that query execution is a significant contributor to the overall runtime of the synthesizer, but many of these queries tend to be the same. Hence, the framework first clusters all steps with the same expansion query, then executes that query once and afterward dispatches to all steps. The framework also manages a blackboard that it passes to each step. The blackboard is a shared data structure that allows various steps to communicate with each other and incrementally build up the algorithm model. Finally, the framework also realizes the graph traversal as such, with the help of the expansion queries, in a breadth-first manner.

A configuration must be provided to select the types of queries that the synthesizer supports. It consists of a configuration per sweep, an ordered list of steps to be applied during the graph expansion and graph traversal, and the order and direction of these sweeps. As an example, a synthesizer for the FPK problem only requires a single sweep as dictated by physics and evident by Algorithm 1, whereas a hybrid dynamics solver demands three sweeps.

#### 5.1.1 Graph expansion by example

**FIGURE 5:**
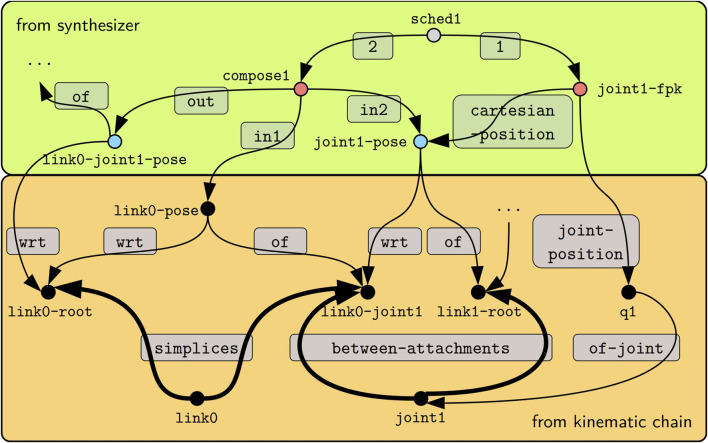
Given the kinematic chain model from Listing 1 (lower orange box), the synthesis step for the FPK algorithm emits a model (upper green box) of the data structures indicated by blue circles (see Listings 2 and 3), the operators indicated by red circles (see Listing 4), and the schedules indicated by gray circles. Edges are labeled by gray panels. The thick edges show how the query in Listing 5 traverses the graph from the start node link0-root (the ?parent) to the link1-root node (the ?child).

We use the FPK solver to exemplify the synthesis in Figure 5. This figure shows an excerpt of a kinematic chain model in the lower box, which is a visual representation of the models from Listings 1 and 2 with an additional joint position q1 composed on top.

As a first step, the synthesizer determines the traversal, that is, which parts of the graph to visit and in which order. Most computations in the kinematics and dynamics solvers propagate quantities between “local” root frames on adjacent links. The SPARQL query in Listing 5 identifies such frames by a transition over a link and over a joint. Assume that the traversal starts at the frame link0-root. This is then the ?node argument passed to the expansion query. Hence, the query tries to follow the geom-ent:simplices first in the “inverse” direction, as indicated by the caret, which would bring it to the link0 node, and then in the “forward” direction so that it arrives at both the link0-joint1 node *and* back at the link0-root node. Next, the FILTER statement eliminates the link0-root node. With the same logic applied to the kc-ent:between-attachments edges, the traversal arrives at the link1-root, which is designated as ?child. Line 4 finally returns any found child (and also the original input node as the parent) as a result of the query.

Listing 5 SPARQL query for frame-to-frame traversal expansion.

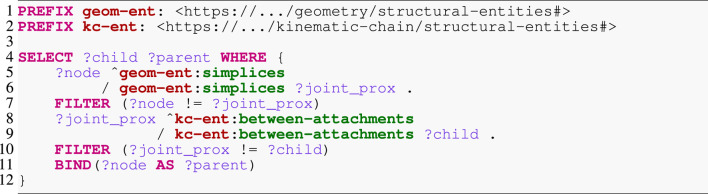

#### 5.1.2 Graph manipulation by example

Next, we investigate how the position propagation step (Algorithm 1 Line 2) manipulates the graph. At first, the step registers a set of visitors, or callbacks, with the framework. During this registration procedure, the step declares the conditions for when these visitors should be executed. The conditions include the mandatory expansion query and further optional queries, for instance, to check if the traversal is currently visiting a leaf node. Finally, the step can decide whether to visit the edge, in which case it receives the parent and child as argument, or whether to only visit the child node. In addition to the expansion query from Listing 5, the position propagation step does not declare any further conditions. Furthermore, this step requires access to the parent’s and child’s states, so it employs an “edge visitor.” The step necessitates two passes: a *configuration* pass to instantiate the algorithm’s data structures and a *computation* pass to instantiate the operators and append them to the schedule.

Continuing with the example in Figure 5, we notice that link0-joint1 is eligible for the position propagation step because it has been reached by the expansion query. During the configuration pass, the step obtains handles to the link0-root-to-joint1 pose, the link0-joint1 frame, and the joint position q1 to then emit the two poses link0-joint1-to-link1-root and link0-root-to-link1-root. Additionally, the joint position and all three poses are added to the blackboard. This enables the computation pass to access them and emit two operators. The first maps the joint position to Cartesian space (joint1-fpk). The second composes the pose of the link and the pose over the joint (compose1). Finally, the pass adds both operators to the schedule (sched1). For this example, we have used human-readable identifiers for all created models; however, in the implementation, we have instead opted for randomly generated universally unique identifiers (UUIDs) as defined by Leach et al. (2005).

Listing 6 IR of algorithm.

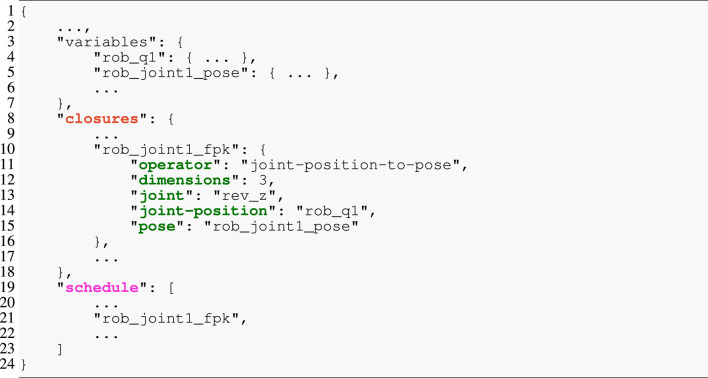

#### 5.1.3 Parallel kinematic chains

Handling parallel kinematic chains requires the interplay of the graph traversal and the visitors. First, during the traversal, each expanded node is assigned a depth, that is, its minimal distance from the node where the traversal started. Then, the visitors feature conditions to handle the different types of edges: *cross edges* connect nodes with the same depth, *forward edges* connect from nodes with lower depth to nodes with higher depth, and *vice versa* for *back edges*. The concrete graph manipulation to be performed for each type of edge is again part of the step. An example in the context of the FPK is to insert a computation that checks, at runtime, if the poses each way around the cycle are consistent. Alternatively, a dynamics solver could insert acceleration constraints as described in Section 2.

### 5.2 Code generation

We have implemented the code generator using the StringTemplate^14^ library and its JSON frontend StringTemplate Standalone Tool^15^. StringTemplate enforces the separation of logic from rendering templates and is one of the few template engines that has scientific justifications for its design and the included and excluded primitives (Parr, 2004). A graphical user interface, the “Inspector,” allows visually debugging the generated code by tracing each rendered token back to a template fragment and its input data. Furthermore, the StringTemplate library is extensively used in the ANTLR parser generator (Parr, 2013).

To bridge the gap between the complete graph models and the template engine, we have introduced the IR and its generator. Its objectives are three-fold. First, because the templates are logic-free, the IR generator performs any necessary processing (e.g., filtering strings so that they represent valid identifiers or embedding information for the template into the IR) on the graph model. Second, it transforms the graph into a tree structure by cutting loops and replacing them with symbolic pointers. Finally, it serializes the resulting graph to JSON, as exemplified in Listing 6. The excerpt of an IR model contains (i) the variables, which is a dictionary with all required data structures, their types, sizes, or initial values; (ii) the closures, a dictionary of operators with their connections to data structures as symbolic pointers (joint-position and pose) and additional properties (dimensions and joint); and (iii) the schedule as an ordered list that contains the symbolic pointers to the closures dictionary.

Listing 7 shows an excerpt of a template model for generating C code. The snippet consists of three rules in Lines 1, 9, and 15. As such, these templates align with our objective of composability because (i) *every* rule is labeled by an identifier and, hence, can be referenced, while (ii) higher-level rules dispatch to lower-level rules. This structure mirrors that of parsers for formal languages, but instead of constructing an abstract syntax tree (AST), it renders text from an AST. The top-level application rule accepts several parameters and then defines the to-be-rendered text between the double angle brackets << and >> (single angle brackets < and > contain StringTemplate processing directives). Here, only the two arguments closure and schedule are shown that align with the IR from Listing 6. The application consists of the program’s main function, which first defines and initializes all required variables (not shown) from the algorithm model’s data structures and then emits the function calls. Line 4 iterates over the schedule and applies the statement rule to each entry with an implicit argument of the currently visited entry and the closures dictionary. Any two generated lines will be separated by a line break. The statement shows another StringTemplate pattern: the notation closures. (closure-id) performs a lookup in the closures dictionary with the value of closure-id as key. Here, another dictionary is returned in which the ().operator retrieves the value associated with the operator. The directive ({rule-id}) (…) then dispatches to the rule rule-id, which is the joint-position-to-pose in this example. This last rule finally renders the function call with the provided arguments.

Listing 7 StringTemplate excerpt for generating the solver implementation. Colors align with the IR from Listing 6.

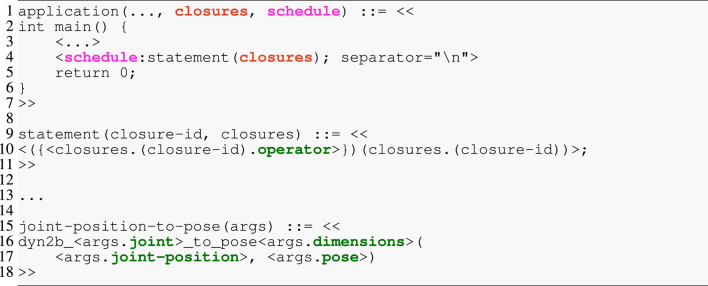

The real implementation separates the top-level *application* template from the reusable and domain-specific *fragments*. We also see that fragments relate to different domains, such as the algorithm model (statement rule) or the kinematics model (joint-position-to-pose rule), and are, hence, located in separate files. As a result, we can efficiently compose a variety of applications by relying on StringTemplate’s import feature to include only the necessary fragments in a top-level template.

### 5.3 dyn2b: support library for computational building blocks

We have also implemented a C software library called dyn2b that realizes the numerical computations for kinematics and dynamics solvers at runtime. dyn2b is designed for composability in that it only provides pure functions at the granularity required by the synthesizer and code generator. First, pure functions are free from side effects, which means that any state must be passed into the functions as explicit arguments to allow for their arbitrary, even reentrant, execution. Second, this design prevents the state from remaining hidden behind a private interface. All too often, algorithm or function developers cannot foresee the context in which their artifacts will be used and, hence, should not introduce preliminary decisions to hide the state. As an example, we have noticed this limitation in the development of an online identification procedure for dynamic parameters that relied on the KDL. Here, the inertial parameters are hidden inside a class that prevents them from being updated using an estimator. Most solver libraries, including RBDL and Pinocchio, already follow such a design that avoids encapsulation: the kinematic chain’s model and/or the solver’s state live in separate yet pre-defined data structures that are publicly accessible. Third, the separation of data from the computations enables (i) the optimization of the data layout for the hardware at hand, including the order of the data structures and their alignment to memory boundaries or cache lines; (ii) state persistence, for instance, by streaming part of the state to a database; or (iii) instrumentation of that state at specific points in time.

The provided functions mainly comprise 3D-Cartesian space kinematics and dynamics to propagate and accumulate the compact representation (cf. Featherstone (2008)) of screws and inertia and functions that map between joint space and Cartesian space for revolute and prismatic joints. In contrast to Featherstone (2008), we do not distinguish between velocity vectors and force vectors but only implement a generic set of functions for screws. The reason is that the type checking, including the checking of additional semantic constraints, is performed on the model level; the code generator then only dispatches to the correct numerical functions. The screw operators act on collections of screws instead of individual screw vectors so that they can efficiently handle the multiple instances of motion drivers. dyn2b explicitly excludes functions for highly variable domains, such as operators associated with more sophisticated joints, stiffness, and damping due to their non-linear behavior, numerical integrators, or trajectory generators. All of these warrant their custom set of models, tools, and software libraries.

dyn2b is compatible with and is built upon the Basic Linear Algebra Subprograms (BLAS) and Linear Algebra Package (LAPACK) libraries. For now, we rely on the very much unoptimized Netlib reference implementations of both libraries. Hence, a significant performance improvement may be possible with a (highly localized) change to a dedicated BLAS implementation for small-scale linear algebra. Here, the role of a general-purpose language compiler is to generate efficient *numerical* code by program-level optimizations such as code inlining, vectorization, constant propagation, or introducing platform-level details, including calling conventions, instruction scheduling, register allocation, and machine code generation.

### 5.4 Steps for dynamics solvers

Analogous to the synthesis and code generation example above, we have also realized the building blocks for further kinematics solvers up to the acceleration level as well as dynamics solvers. Here, we outline the main challenges and policies that we address in our implementation for such solvers.

The main difference for traversing the graph during the inward sweep relates to the handling of leaves, loop-closures, and branching points. We have already discussed the case of an unconditional edge visitor in Section 5.1.2. In contrast, to handle leaves, a step registers a conditional node visitor with the framework that will be called with only the currently visited node as argument. The condition resembles Listing 5, but (i) is a boolean-valued SPARQL ASK query that (ii) checks whether *no* joint follows the link; that is, it is a leaf. The concrete graph manipulation instructions depend on the quantity or motion driver; for instance, inertial force vectors are initialized to zero vectors, whereas the propagated inertia matrix is initialized with the leaf link’s inertia matrix. As for branching points, such as link 1 in Figure 2A, the synthesis step emits operations to accumulate inertia and force over all children of the currently visited segment. Because a serial connection is a special case of a branched connection, we employ the same steps for both in our synthesis tool. The only difference is that the *synthesized* algorithm contains more data structures and operations for branching points.

Next, we turn to the propagation of motion drivers through the kinematic chain. As an example, Figure 2A depicts two instances of external force motion drivers ( $F_{\mathrm{ext,}3}$ and $F_{\mathrm{ext,}4}$ ). Both instances are propagated inward to arrive at link 1, which now “feels” the propagated effect of both forces as $F_{\mathrm{ext,}3}^{\prime }$ and $F_{\mathrm{ext,}4}^{\prime }$ . Traditional solvers would accumulate their effect by adding both forces to minimize the overall number of force variables and, hence, maximize the computational efficiency of the solvers. In contrast, following our recent work (Schneider and Bruyninckx, 2019), the steps for the inward sweep decompose the kinematic chain’s state by propagating all forces and their instances in separation. Then their combined effect at link 1 can be represented as a list: $\left(\begin{array}{cc} F_{\mathrm{ext,}3}^{\prime } & F_{\mathrm{ext,}4}^{\prime } \end{array}\right)$ . In other words, accumulation here means to append. In this setting, it is the role of the synthesis steps to perform the bookkeeping of individual, propagated forces, which includes (i) tracking the sizes of the lists per segment so that their memory can be pre-allocated; (ii) computing the indices into the lists so that each force can be found; and (iii) symbolically associating each propagated force with its original motion driver. For a human consumer, similar names establish the link to the original motion driver, but in the models, a separate relation facilitates the traceability of propagated forces to their original motion specification.

On the one hand, the decomposition during the inward sweep is computationally more expensive than the inward sweep in traditional solvers. On the other hand, it also enables the flexible recomposition of the motion drivers during the final outward sweep or solver sweep. Examples include (i) weighing or prioritizing motion drivers with respect to each other; (ii) avoiding actuator saturation by scaling down some motion drivers in accordance with the motion specification; or (iii) using the decomposed state in model-based controllers (MPC).

## 6 Case study

The objective of the case study is multi-fold. First, it demonstrates the algorithm synthesis and code generation from composable models. Second, it shows the iterative and incremental modeling and development of a concrete application together with its integration into a real robot. Third, it provides evidence of the models’ composability because the application is composed of the solvers’ algorithm models. Finally, it demonstrates compositionality by performing semantic algorithm manipulation. The case study follows the code-centric tutorial that is available together with the toolchain. The objective is to compose a controller and a robot interface onto a recursive Newton–Euler algorithm (RNEA). Afterward, we systematically inject instrumentation operations into the resulting algorithm. Our target platform is a Kinova Gen3^16^ manipulator for which we have created composable models in JSON-LD^17^.

To synthesize the RNEA, we configure the toolchain with two sweeps. The first sweep realizes the outward propagation and accumulation of poses, twists, and acceleration twists. The second sweep realizes the inward propagation of Cartesian-space inertial forces that compensate for gravity and velocity-dependent accelerations. The inward sweep also computes the joint-level control torques associated with the inertial forces. Both sweeps are built from the available steps and code generator fragments that we have explained above. In practice, a robot will have to synthesize (part of) an application whenever the graph changes structurally. For traditional solutions that rely on manually implemented solvers, this high variability leads to a combinatorial explosion for even moderately sized applications, which makes it challenging to design and verify the developed software in advance. Hence, we tackle such problems with our toolchain to synthesize and generate correct-by-construction solvers from verifiable specifications.

### 6.1 Damping controller and robot interface

The first extension is a Cartesian-space motion controller. Its role is to realize the robot’s behavior over a longer time span as contrasted with the solver, which only realizes the instantaneous mappings of force and motion inputs to the control commands of the kinematic chain’s actuators. For the case study, we have opted to demonstrate the controller attachment using a simple damping controller. The controller’s objective is to move the robot’s end-effector while limiting the maximum velocity $v_{\max}$ and maximum force $f_{\max}$ that it could exert on its environment. The controller computes the commanded force as shown in the following Equation 1.

$$f_{\mathrm{cmd}}=f_{\max}-\frac{f_{\max}}{v_{\max}}v_{\mathrm{msr}}$$ (1)

**FIGURE 6:**
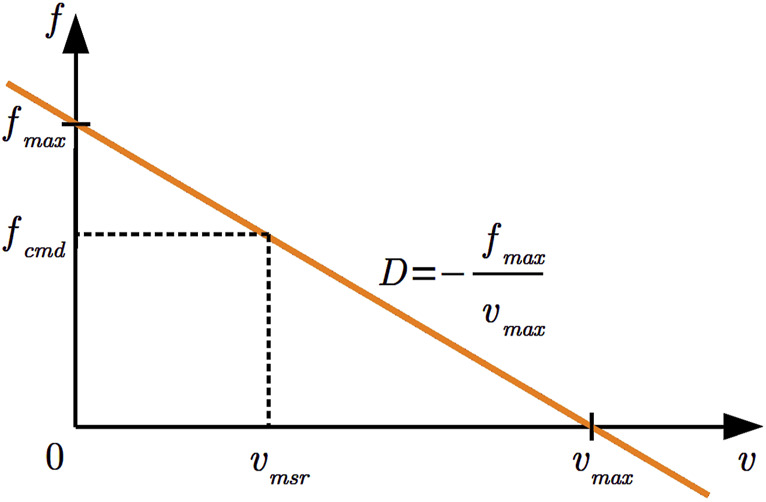
Depiction of the damping controller’s behavior. The linear damping D is computed from a given maximum velocity $v_{\max}$ and a maximum force $f_{\max}$ , as indicated by the orange line. The force to command the robot $f_{\mathrm{cmd}}$ depends on the measured velocity $v_{\mathrm{msr}}$ . In free space, the force accelerates the robot to maximum velocity, whereas in a rigid contact, the robot does not move while still limiting the commanded force.

In this equation, the damping is the intermediate term $D=-\frac{f_{\max}}{v_{\max}}$ that is scaled by the currently measured velocity $v_{\mathrm{msr}}$ . Intuitively, this means that the controller (i) at $v_{\mathrm{msr}}=0$ commands the maximum force so that the robot can accelerate if it is not impeded; (ii) at $v_{\mathrm{msr}}=v_{\max}$ commands no force so that the robot does not accelerate further; and (iii) otherwise linearly interpolates between both cases with D as the proportionality factor. This behavior is depicted in Figure 6. For this case study, we have connected the controller to the robot’s linear upward motion (as seen by the world frame). Hence, the robot always tries to move to a fully stretched-out configuration (a workspace singularity) but can manually be displaced. When the operator holds the robot in place, they feel the robot “pushing” upward with the maximum configured force. If they move it upward too fast (beyond the configured maximum velocity), they feel the robot actively counteracting by braking. An alternative application of this controller is to bring the robot into safely controlled contact with its environment and then align it without relying on additional exteroceptive sensors. As a second extension, we have modeled and implemented a robot interface. The robot interface model attaches to the various joint-level quantities to read joint positions and joint velocities from the robot’s sensors and to command torques to the actuators. The solver handles these attachments by a step to read measurements in the first sweep and another step to write commands in the last sweep. We support two backends: robif2b ^18^ to operate the real robot and a simple simulation that provides fixed measurements while printing joint-level commands to the terminal.

### 6.2 Semantic algorithm manipulation

The algorithm model already enables simple queries to gather statistics about an algorithm or even a complete application. A simple example is to count the number of functions or data structures of a particular type. This may provide insights into the expected performance or memory requirements of the algorithm. However, more interesting queries concern the instrumentation of the running software. To this end, Listing 8 demonstrates a complicated query that inserts a logger into an existing schedule. Apart from the prefix definition at the top, the query consists of three main parts: DELETE to remove elements from the RDF graph, INSERT to extend that graph, and WHERE to localize the deletion and insertion points. We start with the latter, which has the goal of finding an operation ?op of type Damping (Line 19) with an input property velocity-twist and an output property wrench. This operation is part of the ?schedule’s trigger chain (Line 15), the totally ordered collection of operations. RDF represents such a collection as a singly linked list, a set of anonymous nodes that (i) point to the concrete content (here, the operations) via the first property; and (ii) are linked among each other by the rest property that points to the next node. The traversal of this list reads as follows: (i) start at the ?schedule node; (ii) follow one step along the trigger-chain property; (iii) follow an arbitrary number of steps (indicated by the asterisk) along the rest property to visit any list node; and (iv) for each of these nodes, follow to the content via the first property to an operation ?op that must satisfy the above constraints. The query also creates a new UUID as an identifier for the logger (Line 22). The statement in Line 9 instantiates the new logger with a single property quantity that represents an ordered list—indicated by the parentheses—of data structures to log. Notice that apart from the twist and wrench, we also log the joint position of the manipulator’s second joint q2 that experiences the greatest displacement. Finally, Lines 7, 11, and 17 represent, respectively, the cutting, splicing, and bookkeeping of inserting the logger at the correct location into the linked list.

Listing 8 SPARQL update query to insert a logger into a schedule.

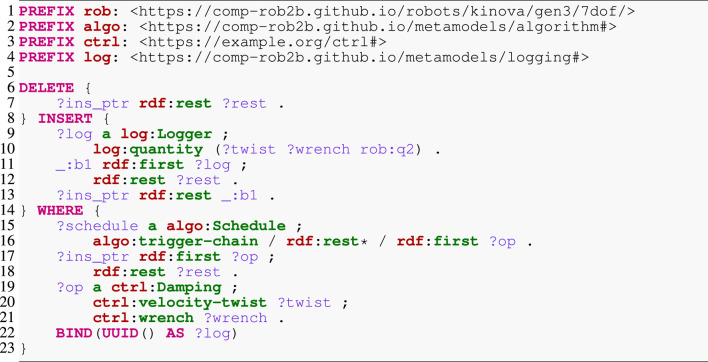

**FIGURE 7:**
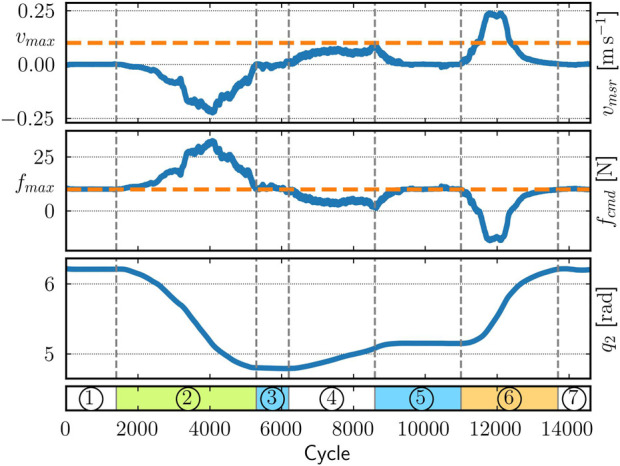
Visualization of the logged data recorded on the real robot. The plot shows the relationship between the end-effector's measured upward velocity (top), the upward control force (middle), and the second joint's position (bottom). The seven labeled phases comprise the robot being fully stretched out (1 and 7), manually pushed downward (2) and upward (6) or held in place (3 and 5) by a human operator, and moving up without contact (4). The controller is configured with *v_max_* = 0.1 m s^−1^ and *f_max_* = 10 N.

For demonstration purposes, we have implemented a simple backend for this logger model that, at runtime, writes the data into a comma-separated value file. The drawback of this approach is that it potentially introduces high amounts of jitter into the real-time control loop. Hence, a more sophisticated approach would rely on a realtime-capable communication infrastructure, such as ring buffers, to send the data to a dedicated log writer. Figure 7 depicts part of this logged data as recorded from the real robot that is executing the above application while intermittently being impeded by a human operator (indicated by colored segments).

The robot starts in a stretched-out configuration, a workspace boundary, so that it points vertically upward. Because it cannot move further in that direction, the controller commands a maximum force of $f_{\max}$ = 10 N. Next, the operator physically displaces the robot until it is parallel to the ground. During this second phase, we see that the robot moves faster than $v_{\max}$ = 0.1 m $s^{-1}$ and, hence, the controller counteracts with a control force greater than $f_{\max}$ . After releasing the arm, in the fourth phase, its velocity gradually increases so that the commanded force reduces. Then, the operator pushes the robot upward so that it exceeds $v_{\max}$ . Here, the controller actively brakes to counteract that upward motion. Not surprisingly, due to the affine control law (scaling and translation), the control command is similar to the measured velocity. Finally, the arm reaches the initial, stretched-out configuration again.

## 7 Discussion

A major inspiration for the toolchain originates from the *strategic programming* paradigm (Lämmel et al., 2003). Although mostly targeted at tree structures, its objective is to separate the *traversal control* from the *logic* that is applied at visited nodes. Here, the traversal strategies are composed of atomic traversal steps and higher-order functions that are called *combinators*. Especially *adaptive programming*, as found in the DJ library (Orleans and Lieberherr, 2001), employs a similar approach as our toolchain by defining graph visitors and their declarative traversal specifications that are then dispatched on a graph of Java objects. With those insights, we refactored our synthesis tool by separating the graph traversal, the synthesis steps, and the blackboard that contains the shared state. Additionally, we realized the query declaration and implemented the caching to reduce the amount of overall query executions.

As shown in the high-level architecture diagram in Figure 4, the individual tools rely on explicit models instead of language-specific APIs. On the one hand, this design allows each tool to be realized with the most suitable programming language and choice of software libraries. On the other hand, also the model representation between two tools can be changed if the producing and consuming tools are adapted. In either case, such a change remains very localized. Here, we will review further variation points and their associated one-to-many mappings with respect to model representation, graph querying languages, template engines, and the execution backend.

The same models can be serialized in various interchange formats, with XML (Bray et al., 2006) and JSON as the most common options. Two models can even be equivalent semantically yet differ structurally. For instance, a RigidBody constraint could be part of an entity’s type or, alternatively, tagged to that entity by a relation. In any case, if the models represent the same information, they can always be model-to-model transformed into each other. In the Semantic Web, the Web Ontology Language (OWL) defined by Bock et al. (2012) provides multiple concepts to perform such transformations.

The decision to use SPARQL was mainly driven by its support for declarative graph matching and its vendor-independent and mature standard, together with the availability of RDFLib. RDFLib eased the integration with our Python toolchain, in particular, due to the in-memory, in-process database that avoids a dedicated database setup. However, in our prior work (Hochgeschwender et al., 2016), we also employed the declarative graph query language Cypher (Francis et al., 2018) that originates from the Neo4j database. Cypher supports graph matching and, as of now, is in the process of being standardized. The imperative language Gremlin (Rodriguez, 2015) is another popular alternative for graph querying that supports graph matching.

We prototyped the code generation with the more popular template engine Jinja^19^ in Schneider et al. (2023). Although the integration with the Python-based toolchain was easy, we quickly noticed the problem of interleaving logic with the templates and a tight coupling of the templates with the execution environment. Examples include the injection of Python functions into the templates as processors or filters and exposing the database interface to the templates. Additionally, in Jinja, the entry point is always an “anonymous” template (not a rule as in StringTemplate) that terminates the composition hierarchy at the top. Developer discipline and macros (akin to rules in StringTemplate) can help, but “clean” templates are not enforced.

Especially when the applications grow more complicated, it may be worthwhile to explore different starting points for the algorithm synthesis. Currently, every synthesis execution starts from scratch. On a computer with an Intel Core i7-4790K CPU and 16 GB RAM, the synthesis takes approximately 1.5 s, while the code generator finishes in approximately 0.3 s. However, it is important to note that this is a design-time cost and *not* part of the real-time path during the running application; the generated code itself *is* real-time capable, that is, it always performs a maximum amount of operations, each with a deterministic runtime. Still, when only some models change, it may be computationally more efficient to reuse previously synthesized algorithms and specialize or modify them in a post-processing step. Another variation point comprises the types of generated artifacts. Because the case study is only an excerpt of the overall application, it suffices to generate completely static code. A simple extension to efficiently change the software’s behavior is to expose and adapt some of the data structures at runtime. Another extension is to generate runtime-composable functions, such as the cascades in a controller, which can be hooked into an application-level event loop to be executed at different cycle rates.

There exists an overlap of our approach and toolchain with functionality in the ROS ecosystem. As mentioned above, the structural models in ROS systems are represented in URDF and SRDF. The robot_state_publisher ^20^ and *tf* (Foote, 2013)^21^ packages provide the software to bring these models to life by realizing the runtime behavior. However, only the FPK computation from Algorithm 1 is realized by these packages: the robot_state_publisher evaluates Line 2, whereas *each* instance of a *tf* listener computes Line 4. These two types of computation are tightly coupled to the ROS communication infrastructure that serializes, sends, receives, and deserializes all pose relations, even if the involved nodes run on the same computer. Once all poses have been accumulated, the *tf* listener can answer queries that require the transformation of points or vectors between coordinate frames. *tf* effectively only supports position-level kinematics: twists can only be approximated by discrete differentiation of poses. Acceleration twists, dynamic quantities and their operators, and maps from Cartesian space to joint space remain completely absent. Additionally, the transformation graph in *tf* must always form a tree. This is caused by the stateless publish-subscribe communication: any node can provide new transforms at any point in time so that the *tf* listener must always construct and evaluate the transform graph anew, which requires a tree as an efficient yet limiting data structure. For similar reasons, *tf* does not support ahead-of-time or just-in-time validation to answer questions such as “*Do the frames in a query exist?*” or “*Does the transform graph actually form a tree?*“

Our approach compares favorably to the currently hyped large language models (Vaswani et al., 2017): it is an engineered solution that is explicit in the represented knowledge, which enables explainability. In other words, composable models represent *exactly* what is necessary, nothing more and nothing less. These properties are also required to certify such a toolchain for safety-critical systems.

## 8 Conclusions and future work

In this article, we present the *graph-based solver* pattern that recurs in various seemingly unrelated robotic domains and is the foundation of various efficient algorithms that act on graph structures. We delve into the details of the pattern by performing an in-depth analysis of solvers on kinematic chains. The supplementary material extends the analysis for probabilistic networks and factor graphs, data-flow models, and expression graphs. We complement the composable models with a model-based engineering toolchain to synthesize such algorithms from the algorithmic building blocks: the data structures, pure functions that act on these data structures, and schedules that describe control flows as sequences of functions. A core element of this toolchain is the synthesizer that accommodates various concerns, including (i) multiple, structured traversals over potentially cyclic graphs; (ii) dispatching computations at specialized node types, both in terms of the graph structure (e.g., at branch nodes, leaf nodes or cycle edges) but also the domain-specific semantics (e.g., at geometric frames, rigid bodies, or kinematic joints); (iii) the algorithm management such as performing memory allocation or triggering computations; and (iv) the incremental construction of the overall algorithm where the operations must have access to a prior *state* from the same sweep or previous sweeps. The synthesizer is an application of higher-order, graph-based reasoning that relies on established standards and mature software libraries. We generate correct-by-construction code from the synthesized algorithm that is complemented by a low-level numeric library to perform the computations required for kinematics and dynamics solvers of rigid-body systems. In a case study, we evaluate our approach on a real robot and demonstrate how the explicit algorithm model facilitates semantic algorithm manipulation.

The proposed approach paves the way to have models for the robot’s complete life-cycle, including runtime aspects of the system. Hence, as future work, we foresee the exploitation of *all* of the above models so that robots can adapt their software themselves even at runtime. To this end, we have already developed a proof-of-concept tool using the llvmlite^22^ library, a Python interface to LLVM’s^23^ just-in-time (JIT) compiler. Additionally, we plan to apply and extend the models and the tools to more complicated applications involving multi-robot systems that must cooperate in challenging manipulation tasks. It is in such systems that the models will pay off the most, due to the complicated robot models and world models that are connected by task or motion descriptions. Here, the graph structure helps in coordinating and configuring a wide range of algorithms, including monitors, controllers, or estimators that are associated with the many relations in the graph.

## Data Availability

The code and models for this article can be found in the following repositories: Synthesis tool and code generator templates: https://github.com/comp-rob2b/kindyngen. Metamodels that define themodel semantics: https://github.com/comp-rob2b/metamodels. Models for the Kinova Gen 3manipulator: https://github.com/comp-rob2b/robot-models. Kinematics and dynamics software library: https://github.com/comp-rob2b/dyn2b.

## Footnotes

1
http://www.orocos.org/kdl

2
http://www.orocos.org/bfl

3
https://www.mathworks.com/products/control.html

4
https://github.com/stack-of-tasks/dynamic-graph

5
https://gstreamer.freedesktop.org/

6 By “dispatching,” we mean to execute or trigger a computation. It resembles dispatchers in operating systems [cf. (Tanenbaum and Bos, 2014)].

7 A metamodel is a model that represents the constraints that a concrete model must satisfy, structurally and semantically, in order to be a “well-formed model” in the context of the application domain the model is designed for.

8
http://wiki.ros.org/urdf

9
http://wiki.ros.org/srdf

10 A counterexample is the rhetoric *metaphor* where the *literal meaning* deviates from the implied, *figurative meaning*.

11
https://w3c.github.io/json-ld-syntax/#relationship-to-rdf

12 This terminology originates from the microblx framework (Klotzbuecher and Bruyninckx, 2013).

13
https://rdflib.dev/

14
https://www.stringtemplate.org/

15
https://github.com/jsnyders/STSTv4

16
https://www.kinovarobotics.com/product/gen3-robots

17
https://github.com/comp-rob2b/robot-models

18
https://github.com/rosym-project/robif2b

19
https://palletsprojects.com/p/jinja/

20
http://wiki.ros.org/robot_state_publisher

21 We use *tf* and *tf2* synonymously.

22
http://llvmlite.pydata.org/

23
https://www.llvm.org/
