# Supplementary material for: Semantic composition of robotic solver algorithms on graph structures
Source: Front Robot AI. 2025 Jan 29;11:1363150. doi: 10.3389/frobt.2024.1363150 (PMC11813742; doi:10.3389/frobt.2024.1363150)
Supplement: Supplementary file 1 [file DataSheet1.pdf]

frontiers

Supplementary Material

1 PROBABILISTIC NETWORKS AND FACTOR GRAPHS

Bayesian networks are among the most commonly used graphical models for probabilistic reasoning in robotics. They are directed acyclic graphs (DAG) that encode random variables as probability distributions via the nodes and direct conditional dependencies as edges between the nodes. Factor graphs as exemplified in Figure S1 generalize Bayesian networks by representing a factorization of an arbitrary function. Structurally, they represent variables *and* factors between these variables. Additionally, the graph structure is undirected so that the factors represent constraint relations[1] between their arguments; it is the queries on top of the graph that introduce directionality.

Variable nodes in Bayesian networks support various types of discrete and continuous probability distributions. Factor graphs lift this restriction and allow for arbitrary variable types to be encoded in the nodes (Loeliger, 2004). Examples can be found in factor graphs for SLAM problems (Dellaert and Kaess, 2017), dynamics solvers for kinematic chains (Xie and Dellaert, 2020), or (equality-constrained) linear-quadratic regulators (Yang et al., 2021).

Assuming that the factor graph in Figure S1 does represent a Bayesian network, we may be interested in computing a so-called marginal function such as $\bar{f}_4(x_4) = p(x_4|x_1, x_2, x_3)$: what is the posterior probability of observing x_4 given the evidence x_1, x_2 and x_3? Efficient approaches for solving such queries rely on dynamic programming (Bellman, 1957). A top-down application, starting at the variable for which the posterior probability is queried (here x_4), leads to the variable elimination algorithm (Russell and Norvig, 2010) which is equivalent to solving the expression on the right-hand side of Equation (S1).

[1] A constraint relation $\dot{\boldsymbol{X}}_A - {}^A\boldsymbol{X}_B\,\dot{\boldsymbol{X}}_B = \mathbf{0}$ can lead to either an assignment $\dot{\boldsymbol{X}}_A \leftarrow {}^A\boldsymbol{X}_B\,\dot{\boldsymbol{X}}_B$ if $\dot{\boldsymbol{X}}_B$ is given and $\dot{\boldsymbol{X}}_A$ is queried for, or $\dot{\boldsymbol{X}}_B \leftarrow {}^A\boldsymbol{X}_B^{-1}\,\dot{\boldsymbol{X}}_A$ in the opposite case.

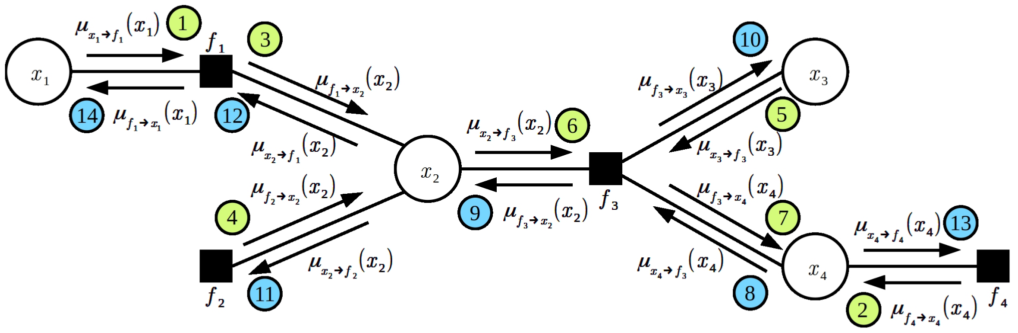

Figure S1. Structurally, the factor graph of the function $\bar{f}(x_1, x_2, x_3, x_4) = f_1(x_1, x_2)f_2(x_2)f_3(x_2, x_3, x_4)f_4(x_4)$ consists of variables (white circle) and factors (black boxes). Message passing imposes behavior on the graph by performing computations at graph nodes and sending messages μ (indicated by arrows) between the nodes. Finally, a schedule (numbers in circles) asserts an ordering constraint on the computations, with green circles for the inward propagation and blue circles for the outward propagation of messages (x_4 is assumed to be the root).

$$\bar{f}_4(x_4) = f_4(x_4)\left(\sum_{x_2,x_3} f_3(x_2,x_3,x_4) f_2(x_2)\left(\sum_{x_1} f_1(x_1,x_2)\right)\right) \tag{S1}$$

However, variable elimination only answers a single query: asking for a different marginal function amounts to forming another expression and solving it from scratch. Hence, a bottom-up application of dynamic programming that starts at the leave nodes and decomposes the network's state to answer multiple queries is preferable. The resulting algorithm is known under the generic name of *message passing*, belief propagation (Pearl, 1982) in the context of Bayesian networks, or the sum-product algorithm for factor graphs. To compute an answer to the above query, message passing proceeds as shown in Figure S1. First, it selects an *arbitrary* variable as the root, here x_4. Then it propagates messages inward from the leaves to the root and afterwards outward from the root to the leaves. Finally, answers can be computed for any variable by multiplying all incoming messages, for example $\bar{f}_4(x_4) = \mu_{f_4 \rightarrow x_4}(x_4)\mu_{f_3 \rightarrow x_4}(x_4)$.

Pearl (1988) provides an example of iterative computations in graphical models in the context of answering queries that are more complicated as the example above. First, message passing solves for the given network. Next, the query is represented as additional nodes that are connected to a subset of that network. Then, the query can be solved by only updating the newly-added nodes while keeping the state of the original network unchanged. This approach can be seen as caching along the "network dimension", i.e. the network retains state when introducing new nodes. It is also possible to perform caching along the "time dimension" by retaining state over several message-passing invocations. As an example, a Kalman filter for outdoor localization is fed by two sources: odometry that provides measurements at higher frequencies and a Global Positioning System (GPS) sensor running at lower frequencies. Then, multiple prediction steps of the Kalman filter using the odometry can be executed for each correction step that relies on the GPS instead. Consequently, distinct parts of the Kalman filter execute at different frequencies.

To connect a graphical model to an encompassing application, it is composed or extended with decision nodes to provide inputs to the network and utility nodes that return feedback to the application (Russell and Norvig, 2010). A simple example of the latter case is a monitor that trigger when some variables have crossed a threshold with a sufficiently high certainty. Such extended networks are also known as influence diagrams (Howard and Matheson, 2005; Jensen and Nielsen, 2007; Kjærulff and Madsen, 2008).

2 DATA-FLOW PROGRAMMING

Data-flow programming is the domain of a variety of block-port-connector (BPC) models or block diagrams for software architectures (Scioni et al., 2016). This includes component-based robotic software frameworks (RSF) (Shakhimardanov et al., 2010; Brugali and Scandurra, 2009; Brugali and Shakhimardanov, 2010) such as the Robot Operating System (ROS) (Quigley et al., 2009) or the Open Robot Control Software (OROCOS) (Bruyninckx et al., 2003) that are among the most prominent data-flow programming approaches in robotics. RSFs facilitate the easy reuse of existing software components, their flexible integration into and iteration of new applications, as well as the distribution of such components in a computer network. The components usually map to processes or threads and, thus, rely on the operating system for scheduling the computations. Most of these frameworks rely on semi-formal, implicit models. Nevertheless, these models have already facilitated the development of a variety of tools for the introspection

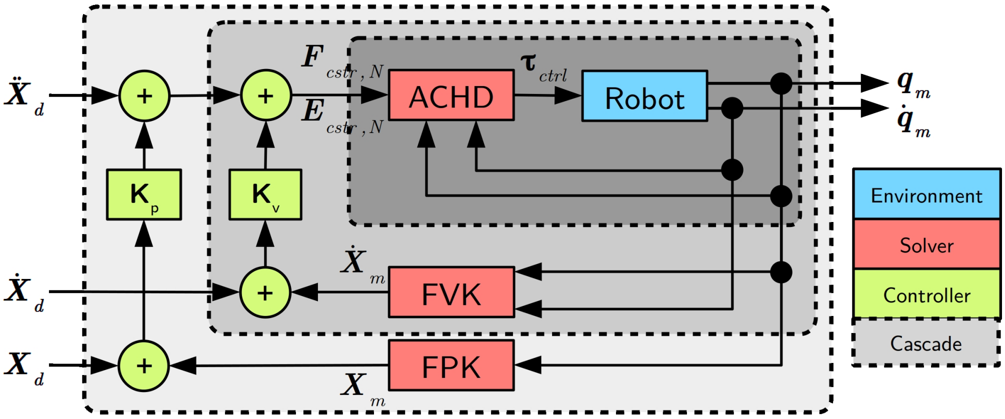

Figure S2. Example of a cascade control diagram where the innermost loop (gray, dashed box) for computing joint control torques $\boldsymbol{\tau}_{ctrl}$ runs at the highest frequency, followed by the intermediate velocity loop $\dot{\boldsymbol{X}}$ and the outer position loop $\boldsymbol{X}$ at subsequently lower frequencies. $\boldsymbol{K}_p$ and $\boldsymbol{K}_v$ are gains that act on the position error and velocity error, respectively. The red boxes represent solvers for the forward position kinematics (FPK), forward velocity kinematics (FVK) and acceleration-constrained hybrid dynamics (Vereshchagin, 1989) (ACHD) problems. The latter solver accepts acceleration constraints as a pair of end-effector unit constraint forces $\boldsymbol{F}_{cstr,N}$ and acceleration energies $\boldsymbol{E}_{cstr,N}$.

(e.g. `rosgraph`[2], `rqt_graph`[3], `TaskBrowser`[4]), and instrumentation (e.g. `rosbag`[5], `rqt_bag`[6], `rqt_console`[7]) of running systems. Additionally, they are the basis for interfacing between different RSFs[8]. However, the components' boundaries are also the boundaries of introspection and instrumentation, meaning the tools are inadequate to look into the components.

Figure S2 depicts a generic cascade control diagram of a trajectory-following controller. Such a visual representation is an attractive property of data-flow graphs that can be more intuitive for a human to understand than a purely mathematical or textual representation. Hence, a variety of data-flow programming tools have emerged that also allow the visual design of complicated systems. Commercial tools include, among others, MathWorks' Simulink[9] or Virtual Instruments (VI) in National Instruments' LabVIEW[10]. Node-RED[11] is another example from the "World Wide Web" domain, whereas the Function Block Diagram as defined in (International Electrotechnical Commission (IEC), 2013) is more prominently found in programmable logic controllers.

A further feature of data-flow graphs is to keep the control flow implicit. As an example, many computations in a robot *(i)* require input data from sensors, which usually provide data at different cycle rates; *(ii)* have input-output dependencies with each other, meaning the output from one computation may be consumed by one or more other computations; and *(iii)* are concurrent, in other words they can be executed reentrant. This is exploited in the Stack of Task's `dynamic-graph` to schedule chains of

[2] http://wiki.ros.org/rosgraph
[3] http://wiki.ros.org/rqt_graph
[4] https://docs.orocos.org/ocl/orocos-taskbrowser.html
[5] http://wiki.ros.org/rosbag
[6] http://wiki.ros.org/rqt_bag
[7] http://wiki.ros.org/rqt_console
[8] https://github.com/orocos/rtt_ros_integration
[9] https://www.mathworks.com/products/simulink.html
[10] https://www.ni.com/labview
[11] https://nodered.org/

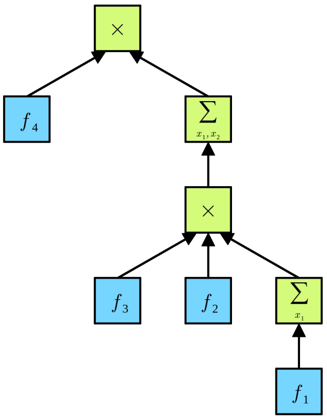

Figure 3a. Expression tree for Equation (S1) with operators in green boxes and variables, constants, or even functions in blue boxes as leaf nodes. Given input at the leaves, the evaluation along the arrows yields the result.

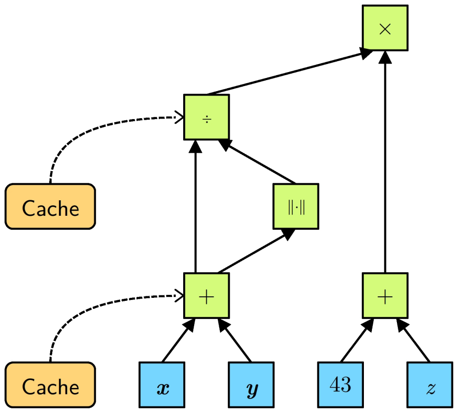

Figure 3b. Expression graph for the expression $\frac{\boldsymbol{x}+\boldsymbol{y}}{\|\boldsymbol{x}+\boldsymbol{y}\|} \times (43+z)$ with $\boldsymbol{x}$ and $\boldsymbol{y}$ as vectors, as well as z as a scalar. Caching (orange boxes) can improve computational performance by avoiding multiple evaluations of common sub-expressions (lower case) or allowing for incremental computation (upper case) — the left branch does not have to be updated when only z changes.

computations dynamically at runtime whenever new sensor data arrives. Consequently, this approach reduces the overall number of required computations, and it enables incremental computations by caching intermediate results.

Structurally, the above data-flow models represent arbitrary computations directly in the graph nodes. The data structures remain implicit as they are indicated only by the edges or arrows between the nodes. Since these edges usually also encode some form of feedback, such diagrams must be represented by generic graphs that can contain directed loops. The graph is executed periodically to realize the intended behavior over time. To this end a single graph traversal suffices that dispatches computations at the nodes. However, that traversal must take care of cutting loops or traversing hierarchically into sub-graphs that are executed at varying frequencies.

3 EXPRESSION TREES AND EXPRESSION GRAPHS

Expression trees are another representation to structure computations. In fact, solvers for tree-structured graphical models can be represented as expressions and, hence, expression trees (Kschischang et al., 2001). As an example, Figure 3a depicts the expression tree for Equation (S1).

Other examples comprise the variety of C++ linear algebra libraries, among them Eigen[12], Blitz++ (Veldhuizen, 1997), uBLAS[13], or Armadillo (Sanderson and Curtin, 2019) which use so-called *expression templates* (Veldhuizen, 1995; Vandevoorde et al., 2002). This allows them to reify the expression tree of linear algebra operations via template metaprogramming. On the one hand, this enables the lazy evaluation of expressions in C++, meaning that the result is computed at the last possible moment before it is required. On the other hand, the expression can then be optimized before its evaluation, for example, by eliminating temporary and intermediate computations, or memory allocations. Drawbacks of this approach

[12] https://eigen.tuxfamily.org/

[13] https://www.boost.org/doc/libs/release/libs/numeric/ublas/

originate from the fact that templates are an internal DSL of C++. As a result, the models are effectively only accessible at compile time by a capable C++ compiler. Additionally, the resulting code fuses models — the expression tree itself — with the implementation — the computations to evaluate the nodes in the expression tree.

The reification of the expression tree also has important applications in modern machine learning frameworks such as TensorFlow or PyTorch. First, just like in the previous C++ libraries it enables the pre-processing and optimization of the expression, as well as dispatching computations to dedicated and optimized numeric libraries. Second, it allows the computation of gradients of the overall expression via automatic differentiation (Rall, 1981; Griewank and Walther, 2008). This is the foundation of training artificial neural networks with the backpropagation algorithm (Baydin et al., 2017). Third, the amount of data that is propagated through the expression tends to be massive so that even the evaluation of individual operators can be costly. In such a situation, sub-expression may be evaluate in a parallel or distributed manner. This is possible because the computational graph explicitly encodes which sub-expressions — the branches — are independent of each other.

The previous approaches represent computations as a tree structure, i.e. computations do not contain a cycle. However, the same data can be connected to multiple operators. Hence, the combination of operators and data can form a directed-acyclic graph (DAG). The `expression graph` library instead reifies expressions so that also the computations can form a DAG as depicted in Figure 3b. As an additional feature, it introduces a mechanism to explicitly encode which sub-graphs should be cached. This allows for incrementally computing expressions and only evaluate sub-graphs when their input data has changed; as well as reducing the overall number of expression evaluations when branches share sub-expressions.

The main insight is that these libraries construct more or less explicit models of algorithms and at a higher level of abstraction than what a compiler for general-purpose languages can see. As a result, the libraries can programmatically manipulate the models, for example, by introducing domain-specific optimizations or by imposing certain computational policies on them. Schedules consist of up to two traversals. The first sweep evaluates the expression as such from the leaves, where the input data resides, to the root. The second sweep, in the opposite direction, can then evaluate derivatives of the expression with respect to chosen parameters. An expression graph directly encodes the to-be-executed operators, but not their derivatives.

REFERENCES

Baydin, A. G., Pearlmutter, B. A., Radul, A. A., and Siskind, J. M. (2017). Automatic Differentiation in Machine Learning: A Survey. *The Journal of Machine Learning Research* 18, 5595–5637

Bellman, R. (1957). *Dynamic Programming* (Princeton University Press)

Brugali, D. and Scandurra, P. (2009). Component-based robotic engineering (Part I) [Tutorial]. *IEEE Robotics & Automation Magazine* 16, 84–96

Brugali, D. and Shakhimardanov, A. (2010). Component-Based Robotic Engineering (Part II). *IEEE Robotics & Automation Magazine* 17, 100–112

Bruyninckx, H., Soetens, P., and Koninckx, B. (2003). The Real-Time Motion Control Core of the Orocos Project. In *Proc. IEEE International Conference on Robotics and Automation (ICRA)*

Dellaert, F. and Kaess, M. (2017). Factor Graphs for Robot Perception. *Foundations and Trends in Robotics* 6, 1–139

Griewank, A. and Walther, A. (2008). *Evaluating Derivatives: Principles and Techniques of Algorithmic Differentiation* (Society for Industrial and Applied Mathematics), second edn.

Howard, R. A. and Matheson, J. E. (2005). Influence Diagrams. *Decision Analysis* 2, 127–143

International Electrotechnical Commission (IEC) (2013). *IEC 61131-3 – Programmable controllers - Part 3: Programming languages*. Standard

Jensen, F. V. and Nielsen, T. D. (2007). *Bayesian Networks and Decision Graphs* (Springer), second edn.

Kjærulff, U. B. and Madsen, A. L. (2008). *Bayesian Networks and Influence Diagrams: A Guide to Construction and Analysis* (Springer), second edn.

Kschischang, F. R., Frey, B. J., and Loeliger, H.-A. (2001). Factor Graphs and the Sum-Product Algorithm. *IEEE Transactions on Information Theory* 47, 498–519

Loeliger, H.-A. (2004). An introduction to factor graphs. *IEEE Signal Processing Magazine* 21, 28–41

Pearl, J. (1982). Reverend Bayes on inference engines: A distributed hierarchical approach. In *Proc. International Joint Conference on Artificial Intelligence*

Pearl, J. (1988). *Probabilistic Reasoning in Intelligent Systems* (Morgan Kaufmann)

Quigley, M., Gerkey, B., Conley, K., Faust, J., Foote, T., Leibs, J., et al. (2009). ROS: an open-source Robot Operating System. In *IEEE International Conference on Robotics and Automation (ICRA). Workshop on Open Source Software*

Rall, L. B. (1981). Automatic Differentiation: Techniques and Applications

Russell, S. J. and Norvig, P. (2010). *Artificial Intelligence: A Modern Approach* (Pearson), 3 edn.

Sanderson, C. and Curtin, R. (2019). Practical Sparse Matrices in C++ with Hybrid Storage and Template-Based Expression Optimisation. *Mathematical and Computational Applications* 24

Scioni, E., Hübel, N., Blumenthal, S., Shakhimardanov, A., Klotzbücher, M., Garcia, H., et al. (2016). Hierarchical Hypergraphs for Knowledge-centric Robot Systems: a Composable Structural Meta Model and its Domain Specific Language NPC4. *Journal of Software Engineering for Robotics (JOSER)* 7, 55–74

Shakhimardanov, A., Hochgeschwender, N., and Kraetzschmar, G. K. (2010). Component Models in Robotics Software. In *Proc. Performance Metrics for Intelligent Systems Workshop (PerMIS)*

Vandevoorde, D., Josuttis, N. M., and Gregor, D. (2002). *C++ Templates – The Complete Guide* (Addison-Wesley)

Veldhuizen, T. (1995). Expression Templates. *C++ Report* 7, 26–31

Veldhuizen, T. (1997). Scientific Computing: C++ Versus Fortran: C++ has more than caught up. *Dr. Dobb's Journal of Software Tools* 22, 34, 36–38, 91

Vereshchagin, A. F. (1989). Modelling and control of motion of manipulational Robots. *Soviet Journal of Computer and Systems Sciences* 27, 29–38. Originally published in Izvestiia Akademii nauk SSSR, Tekhnicheskaya Kibernetika, No. 1, pp. 125–134, 1989

Xie, M. and Dellaert, F. (2020). *A Unified Method for Solving Inverse, Forward, and Hybrid Manipulator Dynamics using Factor Graphs*. Tech. rep. [online], arXiv:1911.10065v2

Yang, S., Chen, G., Zhang, Y., Choset, H., and Dellaert, F. (2021). Equality Constrained Linear Optimal Control With Factor Graphs. In *Proc. IEEE International Conference on Robotics and Automation (ICRA)*
